# Mobile DNA and the TE-Thrust hypothesis: supporting evidence from the primates

**DOI:** 10.1186/1759-8753-2-8

**Published:** 2011-05-31

**Authors:** Keith R Oliver, Wayne K Greene

**Affiliations:** 1 School of Biological Sciences and Biotechnology, Faculty of Science and Engineering, Murdoch University, Perth W. A. 6150, Australia; 2 School of Veterinary and Biomedical Sciences, Faculty of Health Sciences, Murdoch University, Perth W. A. 6150, Australia

## Abstract

Transposable elements (TEs) are increasingly being recognized as powerful facilitators of evolution. We propose the TE-Thrust hypothesis to encompass TE-facilitated processes by which genomes self-engineer coding, regulatory, karyotypic or other genetic changes. Although TEs are occasionally harmful to some individuals, genomic dynamism caused by TEs can be very beneficial to lineages. This can result in differential survival and differential fecundity of lineages. Lineages with an abundant and suitable repertoire of TEs have enhanced evolutionary potential and, if all else is equal, tend to be fecund, resulting in species-rich adaptive radiations, and/or they tend to undergo major evolutionary transitions. Many other mechanisms of genomic change are also important in evolution, and whether the evolutionary potential of TE-Thrust is realized is heavily dependent on environmental and ecological factors. The large contribution of TEs to evolutionary innovation is particularly well documented in the primate lineage. In this paper, we review numerous cases of beneficial TE-caused modifications to the genomes of higher primates, which strongly support our TE-Thrust hypothesis.

## Introduction

**Figure 1:**
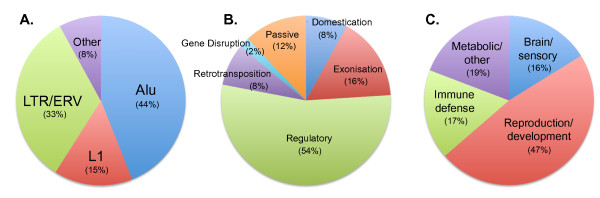
**Summary of the effect of TEs on primate evolution**. **(A) **Transposable elements (TEs) implicated in the generation of primate-specific traits. **(B) **Types of events mediated by TEs underlying primate-specific traits. Passive events entail TE-mediated duplications, inversions or deletions. **(C) **Aspects of primate phenotype affected by TEs. Based on the published data shown in Tables 3 to 6.

Building on the groundbreaking work of McClintock [1] and numerous others [2-14], we further advanced the proposition of transposable elements (TEs) as powerful facilitators of evolution [15] and now formalise this into 'The TE-Thrust hypothesis'. In this paper, we present much specific evidence in support of this hypothesis, which we suggest may have great explanatory power. We focus mainly on the well-studied higher primate (monkey, ape and human) lineages. We emphasize the part played by the retro-TEs, especially the primate-specific non-autonomous Alu short interspersed element (SINE), together with its requisite autonomous partner long interspersed element (LINE)-1 or L1 (Figure 1A). In addition, both ancient and recent endogenizations of exogenous retroviruses (endogenous retroviruses (ERVs)/solo long terminal repeats (sLTRs) have been very important in primate evolution (Figure 1A). The Alu element has been particularly instrumental in the evolution of primates by TE-Thrust. This suggests that, at least in some mammalian lineages, specific SINE-LINE pairs have a large influence on the trajectory and extent of evolution on the different clades within that lineage.

## The TE-Thrust Hypothesis

The ubiquitous, very diverse, and mostly extremely ancient TEs are powerful facilitators of genome evolution, and therefore of phenotypic diversity. TE-Thrust acts to build, sculpt and reformat genomes, either actively by TE transposition and integration (active TE-Thrust), or passively, because after integration, TEs become dispersed homologous sequences that facilitate ectopic DNA recombination (passive TE-Thrust). TEs can cause very significant and/or complex coding, splicing, regulatory and karyotypic changes to genomes, resulting in phenotypes that can adapt well to biotic or environmental challenges, and can often invade new ecological niches. TEs are usually strongly controlled in the soma, where they can be damaging [16,17], but they are allowed some limited mobility in the germline and early embryo [18-20], where, although they can occasionally be harmful, they can also cause beneficial changes that can become fixed in a population, benefiting the existing lineage, and sometimes generating new lineages.

There is generally no Darwinian selection for individual TEs or TE families, although there may be exceptions, such as the primate-specific Alu SINEs in gene-rich areas [21,22]. Instead, according to the TE-Thrust hypothesis, there is differential survival of those lineages that contain or can acquire suitable germline repertoires of TEs, as these lineages can more readily adapt to environmental or ecological changes, and can potentially undergo, mostly intermittently, fecund radiations. We hypothesize that lineages lacking a suitable repertoire of TEs are, if all else is equal, are liable to stasis, possibly becoming 'living fossils' or even becoming extinct.

TE activity is usually intermittent [23-27], with periodic bursts of transposition due to interplay between various cellular controls, various stresses, *de novo *syntheses, *de novo *modifications, new infiltrations of DNA-TEs (by horizontal transfer), or new endogenizations of retroviruses. However, the vast majority of viable TEs usually undergo slow mutational decay and become non-viable (incapable of activity), although some superfamilies have remained active for more than 100 Myr. Episodic TE activity and inactivity, together with differential survival of lineages, suggests an explanation for punctuated equilibrium, evolutionary stasis, fecund lineages and adaptive radiations, all found in the fossil record, and for extant 'fossil species' [15,28].

TE-Thrust is expected to be optimal in lineages in which TEs are active and/or those that possess a high content of homogeneous TEs, both of which can promote genomic dynamism [15]. We hypothesize four main modes of TE-Thrust (Table 1), but as these are extremes of continuums, many intermediate modes are possible.

**Table 1:** Hypothesized major modes of transposable element (TE)-thrust

Mode	TE activity	TE homogeneity	TE population size	Evolutionary outcome	Type of TE thrust
1	Viable and intermittently active	Heterogeneous	Large	Stasis with punctuation events	Active
			Small	Stasis with punctuation events	Active
2	Viable and intermittently active	Homogeneous	Large	Gradualism with punctuation events	Active and passive
			Small	Stasis with punctuation events	Active
3	Non-viable/Inactive	Heterogeneous	Large	Stasis^a,b^	Minimal^c^
			Small	Stasis^a,b^	Minimal^c^
4	Non-viable/Inactive	Homogeneous	Large	Gradualism^a^	Passive^c^
			Small	Stasis^a,b^	Minimal^c^

^a^Unless new infiltrations or reactivation of TEs occur.

^b^Fossil taxa are a possible outcome of prolonged stasis.

^c^Inactive/non-viable TEs can be exapted in a delayed fashion, which could cause some resumption of active TE-Thrust.

• Mode 1: periodically active heterogeneous populations of TEs result in stasis with the potential for intermittent punctuation events.

• Mode 2: periodically active homogenous populations of TEs result in: 1) gradualism as a result of ectopic recombination, if the TE population is large, with the potential for periodic punctuation events, or 2) stasis with the potential for periodic punctuation events if the TE population is small.

• Mode 3: non-viable heterogeneous populations of TEs, in the absence of new infiltrations, result in prolonged stasis, which can sometimes result in extinctions and/or 'living fossils'.

• Mode 4: non-viable homogenous populations of TEs, in the absence of new infiltrations, can result in: 1) gradualism as a result of ectopic recombination, if the TE population is large or 2) stasis if the TE population is small.

These modes of TE-Thrust are in agreement with the findings of palaeontologists [29] and some evolutionary biologists [30] that punctuated equilibrium is the most common mode of evolution, but that gradualism and stasis also occur. Many extant 'living fossils' are also known.

We acknowledge that TE-Thrust acts by enhancing evolutionary potential, and whether that potential is actually realized is heavily influenced by environmental, ecological and other factors. Moreover, there are many other 'engines' of evolution besides TE-Thrust, such as point mutation, simple sequence repeats, endosymbiosis, epigenetic modification and whole-genome duplication [31-35], among others. These often complement TE-Thrust; for example, point mutations can endow duplicated or retrotransposed genes with new functions [36,37]. There may also be other, as yet unknown, or hypothesized but unconfirmed, 'engines' of evolution.

## Higher primate genomes are very suited to TE-Thrust as they possess large homogeneous populations of TEs

Human and other extant higher primate genomes are well endowed with a relatively small repertoire of TEs (Table 2). These TEs, which have been extensively implicated in engineering primate-specific traits (Table 3; Table 4; Table 5; Table 6), are largely relics of an evolutionary history marked by periodic bursts of TE activity [25,38,39]. TE activity is presently much reduced, but extant simian lineage genomes remain well suited for passive TE-Thrust, with just two elements, Alu and L1, accounting for over 60% of the total TE DNA sequence [21,40,41]. In humans, there are 10 times as many mostly homogeneous class I retro-TEs as there are very heterogeneous class II DNA-TEs [21]. Only L1, Alu, SVA (SINE-R, variable number of tandem repeats (VNTR), Alu) and possibly some ERVs, remain active in humans [42].

**Table 2:** Summary of the major transposable elements (TEs) found in humans

	Family	Percentage of genome	Number in genome	Average length, bp	Maximum length, kb	Viable	Potentially autonomous
Type I: retro-TEs	LTR^a^/ERV^b^	8.3	443,000	510	10	No	Yes (via reverse transcriptase)
	LINE1^c^	16.9	516,000	900	6	Some	Yes (via reverse transcriptase)
	LINE2	3.2	315,000	280	5	No	Yes (via reverse transcriptase)
	Alu SINE^d^	10.6	1,090,000	270	0.3	Yes	No
	MIR^e ^SINE	2.2	393,000	150	0.26	No	No
	SVA^f ^SINE-like composite	0.2	3,000	1,400	3	Yes	No
Type II: DNA-TEs	Many	2.8	294,000	260	3	No	Some (via transposase)

^a^LTR = long terminal repeat

^b^ERV = endogenous retrovirus

^c^LINE = long interspersed nuclear element

^d^SINE = short interspersed nuclear element

^e^MIR = mammalian-wide interspersed repeat

^f^SVA = SINE-VNTR-Alu

**Table 3:** Specific examples of transposable elements (TEs) implicated in primate-specific traits: brain and sensory

TE generated trait	Gene affected	Gene function	TE responsible	Distribution^a^	Type of event	Effect	Tissue expression	Type of TE-Thrust	Reference
	*snaRs*	Cell growth and translational regulation	Alu	Afr. great ape/ human	Domestication	Novel genes	Brain, testis	Active	Parrott and Mathews, 2009 [105]
	*BCYRN1*	Translational regulation of dendritic proteins	Alu	Simian	Domestication	Novel gene	Brain	Active	Watson and Sutcliffe, 1987 [106]
	*FLJ33706*	Unknown	Alu	Human	Domestication	Novel gene	Brain	Active	Li *et al.*, 2010 [107]
Neuronal stability?	*SETMAR*	DNA repair and replication	Hsmar1	Simian	Exonization	Novel fusion gene	Brain, various	Active	Cordaux *et al.*, 2006 [108]
	*Survivin*	Anti-apoptotic/brain development	Alu	Ape	Exonization	Novel isoform	Brain, spleen	Active	Mola *et al.*, 2007 [109]
	*ADARB1*	RNA editing/neurotransmitter receptor diversity	Alu	>Human	Exonization	Novel isoform	Brain, various	Active	Lai *et al.*, 1997 [110]
	*CHRNA1*	Synaptic transmission	MIR^b^	Great ape	Exonization	Novel isoform	Neuromuscular	Active	Krull *et al.*, 2007 [47]
	*ASMT*	Melatonin synthesis	LINE-1^c^	>Human	Exonization	Novel isoform	Pineal gland	Active	Rodriguez *et al.*, 1994 [111]
	*CHRNA3*	Synaptic transmission	Alu	Great ape	Regulatory	Major promoter	Nervous system	Active	Fornasari *et al.*, 1997 [112]
	*CHRNA6*	Synaptic transmission	Alu	>Human	Regulatory	Negative regulation	Brain	Active	Ebihara *et al.*, 2002 [113]
	*NAIP*	Anti-apoptosis (motor neuron)	Alu	>Human	Regulatory	Alternative promoters	CNS, various	Active	Romanish *et al.*, 2009 [114]
	*CNTNAP4*	Cell recognition/adhesion	ERV^d^	>Human	Regulatory	Alternative promoter	Brain, testis	Active	van de Lagemaat *et al.*, 2003 [73]
	*CCRK*	Cell cycle-related kinase	Alu	Simian	Regulatory	CpG island	Brain	Active	Farcas *et al.*, 2009 [86]
Enhanced cognitive capacity/memory?	*GLUD2*	Neurotransmitter recycling	Unknown	Ape	Retrotransposition	Novel gene	Brain	Active	Burki and Kaessmann, 2004 [37]
Altered auditory perception?	*CHRNA9*	Cochlea hair development/ modulation of auditory stimuli	Alu	Human	Deletion	Exon loss	Cochlea, sensory ganglia	Passive	Sen *et al.*, 2006 [62]
Trichromatic colour vision	*OPN1LW*	Cone photoreceptor	Alu	Old World primate	Duplication	Novel gene	Retina	Passive	Dulai *et al.*, 1999 [36]

^a ^> = Maximum known distribution.

^b^MIR = mammalian-wide interspersed repeat

^c^LINE = long interspersed nuclear element

^d^ERV = endogenous retrovirus

**Table 4:** Specific examples of transposable elements (TEs) implicated in primate-specific traits: reproduction and development

TE generated trait	Gene affected	Gene function	TE responsible	Distribution^a^	Type of event	Effect	Tissue expression	Type of TE-Thrust	Reference
Placental morphogenesis	*Syncytin-1*	Trophoblast cell fusion	ERV^b^	Ape	Domestication	Novel gene	Placenta	Active	Mi *et al.*, 2000 [92]
Placental morphogenesis	*Syncytin-2*	Trophoblast cell fusion	ERV	Simian	Domestication	Novel gene	Placenta	Active	Blaise *et al.*, 2003 [93]
	*HERVV1*	Unknown	ERV	Simian	Domestication	Novel gene	Placenta	Active	Kjeldbjerg *et al.*, 2008 [115]
	*HERVV2*	Unknown	ERV	Simian	Domestication	Novel gene	Placenta	Active	Kjeldbjerg *et al.*, 2008 [115]
	*ERV3*	Development and differentiation?	ERV	Old World primate	Domestication	Novel gene	Placenta, various	Active	Larsson *et al.*, 1994 [116]
	*DNMT1*	DNA methylation	Alu	>Afr. great ape	Exonization	Novel isoform	Fetal, various	Active	Hsu *et al.*, 1999 [117]
	*LEPR*	Leptin receptor	SVA	Human	Exonization	Novel isoform	Fetal liver	Active	Damert *et al.*, 2004 [118]
	*IL22RA2*	Regulation of inflammatory responses/interleukin-22 decoy receptor	LTR^c^	Great ape	Exonization	Novel isoform	Placenta	Active	Piriyapongsa *et al.*, 2007 [119]
	*PPHLN1*	Epithelial differentiation/nervous-system development	ERV/Alu/LINE-1^d^	Ape	Exonization	Novel isoforms	Fetal, various	Active	Huh *et al.*, 2006 [120]
	*CGB1/2*	Chorionic gonadotropin	Alu (snaR-G1/2)	Afr. great ape	Regulatory	Major promoter	Testis	Active	Parrott and Mathews, 2009 [105]
	*GSDMB*	Epithelial development	Alu	Ape	Regulatory	Major promoter	Stomach	Active	Komiyama *et al.*, 2010 [121]
	*HYAL4*	Hyaluronidase	LINE-1/Alu	>Human	Regulatory	Major promoter	Placenta	Active	van de Lagemaat *et al.*, 2003 [73]
Placental oestrogen synthesis	*HSD17B1*	Oestrogen synthesis	ERV	>Human	Regulatory	Major promoter	Ovary, placenta	Active	Cohen *et al.*, 2009 [122]
Placental development	*INSL4*	Regulation of cell growth and metabolism	ERV	Old World primate	Regulatory	Major promoter	Placenta	Active	Bieche *et al.*, 2003 [123]
	*DSCR4*	Unknown reproductive function	ERV	Ape	Regulatory	Major promoter	Placenta, testis	Active	Dunn *et al.*, 2006 [124]
	*DSCR8*	Unknown reproductive function	ERV	>Ape	Regulatory	Major promoter	Placenta, testis	Active	Dunn *et al.*, 2006 [124]
	*CGA*	Common subunit of chorionic gonadotropin, luteinizing, follicle-stimulating and thyroid-stimulating hormones	Alu	>Simian	Regulatory	Negative regulation	Placenta, pituitary gland	Active	Scofield *et al.*, 2000 [125]
Globin switching	*HBE1*	Embryonic oxygen transport	Alu	>Human	Regulatory	Negative regulation	Fetal	Active	Wu *et al.*, 1990 [126]
	*GH*	Growth hormone	Alu	>Human	Regulatory	Negative regulation	Pituitary gland	Active	Trujillo *et al.*, 2006 [127]
	*WT1*	Urogenital development	Alu	>Human	Regulatory	Negative regulation	Urogenital	Active	Hewitt *et al.*, 1995 [128]
Efficient placental gas exchange	*HBG1*	Fetal oxygen transport	LINE-1	Old World primate	Regulatory	Tissue-specific enhancer	Fetal	Active	Johnson *et al.*, 2006 [91]
Placental leptin secretion	*LEP*	Metabolic regulatory hormone	LTR	>Human	Regulatory	Tissue-specific enhancer	Placenta	Active	Bi *et al.*, 1997 [129]
	*MET*	Hepatocyte growth-factor receptor	LINE-1	> Afr. great ape	Regulatory	Alternative promoter	Liver, Pancreas, Lung	Active	Nigumann *et al.*, 2002 [71]
	*BCAS3*	Embryogenesis/erythropoiesis	LINE-1	> Afr. great ape	Regulatory	Alternative promoter	Fetal, various	Active	Wheelan *et al.*, 2005 [130]
	*CHRM3*	Synaptic transmission	LINE-1	Human	Regulatory	Alternative promoter	Placenta	Active	Huh *et al.*, 2009 [131]
	*CLCN5*	Chloride transporter	LINE-1	>Human	Regulatory	Alternative promoter	Placenta	Active	Matlik *et al.*, 2006 [132]
	*SLCO1A2*	Organic anion transporter	LINE-1	>Human	Regulatory	Alternative promoter	Placenta	Active	Matlik *et al.*, 2006 [132]
	*CHRM3*	Synaptic transmission	LTR	Human	Regulatory	Alternative promoter	Testis	Active	Huh *et al.*, 2009 [131]
	*IL2RB*	Growth-factor receptor	LTR	>Human	Regulatory	Alternative promoter	Placenta	Active	Cohen *et al.*, 2009 [122]
Placental development	*ENTPD1*	Thromboregulation	LTR	>Human	Regulatory	Alternative promoter	Placenta	Active	van de Lagemaat *et al.*, 2003 [73]
	*MKKS*	Molecular chaperone	LTR/LINE-2	>Human	Regulatory	Alternative promoter	Testis, fetal	Active	van de Lagemaat *et al.*, 2003 [73]
	*NAIP*	Anti-apoptosis	ERV	>Human	Regulatory	Alternative promoter	Testis	Active	Romanish *et al.*, 2007 [133]
	*EDNRB*	Placental development/circulation	ERV	>Human	Regulatory	Alternative promoter	Placenta	Active	Medstrand *et al.*, 2001 [134]
Placental development	*PTN*	Growth factor	ERV	Ape	Regulatory	Alternative promoter	Trophoblast	Active	Schulte *et al. *1996 [135]
	*MID1*	Cell proliferation and growth	ERV	Old World primate	Regulatory	Alternative promoter	Placenta, fetal kidney	Active	Landry *et al.*, 2002 [136]
	*NOS3*	Endothelial nitric oxide synthesis	ERV	>Human	Regulatory	Alternative promoter	Placenta	Active	Huh *et al.*, 2008 [137]
	*GSDMB*	Epithelial development	ERV	Ape	Regulatory	Alternative promoter	Various	Active	Sin *et al.*, 2006 [138]
Placental oestrogen synthesis	*CYP19*	Oestrogen synthesis	ERV	Simian	Regulatory	Alternative promoter	Placenta	Active	van de Lagemaat *et al.*, 2003 [73]
	*AMACs*	Fatty-acid synthesis	SVA	Afr. great ape	Retrotransposition	Novel genes	Placenta, testis	Active	Xing *et al.*, 2006 [139]
	*POTEs*	Pro-apoptosis/spermatogenesis	LINE-1	Ape	Retrotransposition	Novel fusion genes	Testis, ovary, prostate, placenta	Active	Lee *et al.*, 2006 [140]
	*PIPSL*	Intracellular protein trafficking	LINE-1	>Great ape	Retrotransposition	Novel fusion gene	Testis	Active	Babushok *et al.*, 2007 [141]
	*CDYs*	Chromatin modification	Unknown	Simian	Retrotransposition	Novel genes	Testis	Active	Lahn and Page, 1999 [142]
	*ADAM20/21*	Membrane metalloprotease	Unknown	>Human	Retrotransposition	Novel genes	Testis	Active	Betran and Long, 2002 [143]
Placental growth hormone secretion	*GH*	Placental growth hormone	Alu	Simian	Duplication	Novel genes	Placenta	Passive	De Mendoza *et al.*, 2004 [88]
	*Chr19 miRNAs*	Unknown	Alu	Simian	Duplication	Novel genes	Placenta	Passive	Zhang *et al.*, 2008 [144]
Enhanced immune tolerance at fetal-maternal interface	*LGALS13/14/16*	Carbohydrate recognition/immune regulation	LINE-1	Simian	Duplication	Novel genes	Placenta	Passive	Than *et al.*, 2009 [145]
Efficient placental gas exchange	*HBG2*	Fetal oxygen transport	LINE-1	Simian	Duplication	Novel gene	Fetal	Passive	Fitch *et al.*, 1991 [90]

^a ^> = Maximum known distribution.

^b^ERV = endogenous retrovirus

^c^LTR = long terminal repeat

^d^LINE = long interspersed nuclear element

**Table 5:** Specific examples of transposable elements (TEs) implicated in primate-specific traits: immune defence

TE generated trait	Gene affected	Gene function	TE responsible	Distribution^a^	Type of event	Effect	Tissue expression	Type of TE-Thrust	Reference
Soluble CD55	*CD55*	Complement regulation	Alu	>Human	Exonization	Novel isoform	Various	Active	Caras *et al.*, 1987 [146]
Intracellular TNFR	*P75TNFR*	Tumour necrosis factor receptor	Alu	Old World primate	Exonization	Novel isoform	Various	Active	Singer *et al.*, 2004 [147]
Altered infectious-disease resistance?	*IRGM*	Intracellular pathogen resistance	ERV^b^	Afr. Great Ape	Regulatory	Major promoter	Various	Active	Bekpen *et al.*, 2009 [148]
Altered infectious-disease resistance?	*IL29*	Antiviral cytokine	Alu/LTR^c^	>Human	Regulatory	Positive regulation	Dendritic cells, epithelial cells	Active	Thomson *et al.*, 2009 [149]
	*FCER1G*	IgE/IgG Fc receptor/T cell antigen receptor	Alu	Ape	Regulatory	Positive/negative regulation	T cells, basophils	Active	Brini *et al.*, 1993 [150]
	*CD8A*	T cell interaction with class I MHC	Alu	Ape	Regulatory	Tissue-specific enhancer	T cells	Active	Hambor *et al.*, 1993 [151]
Red cell ABH antigen expression	*FUT1*	Fucosyltransferase	Alu	Ape	Regulatory	Alternative promoter	Erythrocytes	Active	Apoil *et al.*, 2000 [96]
	*TMPRSS3*	Membrane serine protease	Alu/LTR	>Human	Regulatory	Alternative promoter	Peripheral blood leukocytes	Active	van de Lagemaat *et al.*, 2003 [73]
Colon Le antigen expression	*B3GALT5*	Galactosyltransferase	ERV	Old World primate	Regulatory	Alternative promoter	Colon, small intestine, breast	Active	Dunn *et al.*, 2003 [152]
Prolactin potentiation of the adaptive immune response	*PRL*	Regulation of lactation and reproduction	ERV	Old World primate	Regulatory	Alternative promoter	Lymphocytes, endometrium	Active	Gerlo *et al.*, 2006 [153]
	*ST6GAL1*	Sialyltransferase	ERV	>Human	Regulatory	Alternative promoter	B lymphocytes	Active	van de Lagemaat *et al.*, 2003 [73]
Vitamin D regulation of cathelicidin antimicrobial peptide gene	*CAMP*	Antimicrobial peptide	Alu	Simian	Regulatory	Vitamin D responsiveness	Myeloid cells, various	Active	Gombart *et al.*, 2009 [98]
	*MPO*	Myeloperoxidase/microbicidal enzyme	Alu	>Human	Regulatory	Thyroid hormone/retinoic acid responsiveness	Myeloid cells	Active	Piedrafita *et al.*, 1996 [154]
Altered infectious-disease resistance?	*IFNG*	Antiviral/immunoregulatory factor	Alu	Old World primate	Retrotransposition	Novel positive regulatory element	Natural killer cells, T cells	Active	Ackerman *et al.*, 2002 [155]
Absence of N-glycolylneuraminic acid/altered infectious-disease resistance?	*CMAH*	*N*-glycolylneuraminic acid synthesis	Alu	Human	Gene disruption	Gene loss	Various	Active	Hayakawa *et al.*, 2001 [104]
	*IRGM*	Intracellular pathogen resistance	Alu	Old and New World monkey	Gene disruption	Gene loss	Various	Active	Bekpen *et al.*, 2009 [148]
Altered malaria resistance?	*HBA2*	Oxygen transport	Alu	>Ape	Duplication	Novel gene	Erythrocytes	Passive	Hess *et al.*, 1983 [156]

^a ^> = Maximum known distribution.

^b^ERV = endogenous retrovirus

^c^LTR = long terminal repeat

**Table 6:** Specific Examples of transposable elements (TEs) implicated in primate-specific traits: metabolic and other

TE generated trait	Gene affected	Gene function	TE responsible	Distribution^a^	Type of event	Effect	Tissue expression	Type of TE-Thrust	Reference
	*RNF19A*	Ubiquitin ligase	Alu	> Human	Exonization	Novel isoform	Various	Active	Huh *et al.*, 2008 [157]
	*BCL2L11*	Pro-apoptotic	Alu	> Human	Exonization	Novel isoform	Various	Active	Wu *et al.*, 2007 [158]
	*BCL2L13*	Pro-apoptotic	Alu	> Human	Exonization	Novel isoform	Various (cytosolic instead of mitochondrial)	Active	Yi *et al.*, 2003 [159]
	*SFTPB*	Pulmonary surfactant	Alu/ERV^b^	Primate	Exonization	Novel isoform	Various	Active	Lee *et al.*, 2009 [160]
Efficiency of ZNF177 transcription and translation	*ZNF177*	Transcriptional regulator	Alu/LINE-1^c^/ERV	> Human	Exonization	Novel isoform	Various	Active	Landry *et al.*, 2001 [161]
Production of salivary amylase	*AMY1s*	Starch digestion	ERV	Old World primate	Regulatory	Major promoter	Salivary gland	Active	Ting *et al.*, 1992 [99]
	*BAAT*	Bile metabolism	ERV	> Human	Regulatory	Major promoter	Liver	Active	van de Lagemaat *et al.*, 2003 [73]
	*CETP*	Cholesterol metabolism	Alu	> Human	Regulatory	Negative regulation	Liver	Active	Le Goff *et al.*, 2003 [162]
Absence of FMO1 in adult liver/altered drug metabolism?	*FMO1*	Xenobiotic metabolism	LINE-1	> Human	Regulatory	Negative regulation in liver	Kidney	Active	Shephard *et al.*, 2007 [163]
	*RNF19A*	Ubiquitin ligase	LTR^d^	> Human	Regulatory	Alternative promoter	Various	Active	Huh *et al.*, 2008 [157]
	*APOC1*	Lipid metabolism	ERV	Ape	Regulatory	Alternative promoter	Various	Active	Medstrand *et al.*, 2001 [134]
	*KRT18*	Epithelial keratin	Alu	> Human	Regulatory	Retinoic acid responsiveness	Various	Active	Vansant and Reynolds, 1995 [77]
	*PTH*	Parathyroid hormone	Alu	> Old World primate	Regulatory	Negative calcium responsiveness	Parathyroid gland	Active	McHaffie and Ralston, 1995 [164]
	*PRKACG*	cAMP signalling/regulation of metabolism	Unknown	> Old World primate	Retrotransposition	Novel gene	Various	Active	Reinton *et al.*, 1998 [165]
	*NBR2*	Unknown	Alu	Old World primate	Duplication	Novel gene	Various	Passive	Jin *et al.*, 2004 [166]
	*LRRC37A*	Unknown	Alu	Old World primate	Duplication	Novel genes	Various	Passive	Jin *et al.*, 2004 [166]
	*ARF2*	GTPase/vesicle trafficking	Alu	Great ape	Inversion	Novel fusion gene	Various	Passive	Jin *et al.*, 2004 [166]
Altered arterial wall function?	*ELN*	Elastin	Alu	> Old World primate/human	Deletion	Exon losses	Various	Passive	Szabo *et al.*, 1999 [167]
Low body mass?	*ASIP*	Energy metabolism/pigmentation	Alu	Lesser ape (gibbon)	Deletion	Gene loss	Various	Passive	Nakayama and Ishida, 2006 [101]

^a ^> = Maximum known distribution.

^b^ERV = endogenous retrovirus

^c^LINE = long interspersed nuclear element

^d^LTR = long terminal repeat

L1 and the primate-specific Alu predominate in simians [21,40,41], and thus strongly contribute to TE-Thrust in this lineage (Figure 1A). The autonomous L1 is almost universal in mammals, whereas the non-autonomous Alu, like most SINEs, is conspicuously lineage-specific, having been synthesized *de novo*, extremely unusually, from a 7SL RNA-encoding gene. The confinement of Alu to a single mammalian order is typical of younger SINEs, whereas ancient SINEs, or exapted remnants of them, may be detectable across multiple vertebrate classes [43]. Alu possesses additional unusual characteristics: extreme abundance (1.1 million copies, occurring every 3 kb on average in the human genome), frequent location in gene-rich regions, and a lack of evolutionary divergence [21,44]. Their relatively high homology is most easily explained as being the result of functional selection helping to prevent mutational drift. Thus, Alus have been hypothesized to serve biological functions in their own right, leading to their selection and maintenance in the primate genome [22]. For example, A-to-I RNA editing, which has a very high prevalence in the human genome, mainly occurs within Alu elements [45], which would seem to provide primates with a genetic sophistication beyond that of other mammals. Alus may therefore not represent a peculiar, evolutionary neutral invasion, but rather positively selected functional elements that are resistant to mutational degradation [46]. This has significance for TE-Thrust, as it would greatly prolong the usefulness of Alus as facilitators of evolution within primate lineages.

Other human retro-TEs include the fossil tRNA mammalian-wide intespersed repeat (MIR) SINE, which amplified approximately 130 Mya [21,47] and the much younger SVA, a non-autonomous composite element partly derived from ERV and Alu sequences, which is specific to the great apes and humans [48]. Like Alus, SVAs are mobilised by L1-encoded enzymes and, similar to Alu, a typical full-length SVA is GC-rich, and thus constitutes a potential mobile CpG island. Importantly, ERVs are genome builders/modifiers of exogenous origin [49]. Invasion of ERVs seems to be particularly associated with a key mammalian innovation, the placenta (Table 4). The endogenisation of retroviruses and the horizontal transfer of DNA-TEs into germlines clearly show that the Weismann Barrier is permeable, contrary to traditional theory.

The DNA-TEs, which comprise just 3% of the human genome, are extremely diverse, but are now completely inactive [21,50]. Although some have been exapted within the simian lineage as functional coding sequences (Table 3; Table 4; Table 5; Table 6), DNA-TEs, it seems, cannot now be a significant factor for TE-Thrust in primates, unless there are new infiltrations.

## TE-Thrust influences evolutionary trajectories

A key proposal of our TE-Thrust hypothesis is that TEs can promote the origin of new lineages and drive lineage divergence through the engineering of specific traits. Ancestral TEs shared across very many lineages can, by chance, lead to the delayed generation of traits in one lineage but not in another. For example, more than 100 copies of the ancient amniote-distributed AmnSINE1 are conserved as non-coding elements specifically among mammals [51]. However, as they often show a narrow lineage specificity, we hypothesize that younger SINEs (with their partner LINEs) may have a large influence upon the trajectory and the outcomes of the evolution within clades, as is apparent with the Alu/L1 pair in primates (Figure 1A). Probably not all SINEs are equal in this ability; it seems that some SINEs are more readily mobilised than others, and when mobilised, some SINEs are more effective than others at facilitating evolution by TE-Thrust. The extremely abundant primate Alu dimer seems to illustrate this. Whereas the overwhelming majority of SINEs are derived from tRNAs, Alus may have proliferated so successfully because they are derived from the 7SL RNA gene [52], which is part of the signal recognition particle (SRP) that localises to ribosomes. Alu RNAs can therefore bind proteins on the SRP and thus be retained on the ribosome, in position to be retrotransposed by newly synthesized proteins encoded by their partner L1 LINEs [53].

Among the primates, the simians have undergone the greatest evolutionary transitions and radiation. Of the approximately 367 extant primate species, 85% are simians, with the remainder being prosimians, which diverged about 63 Mya. Significantly, large amplifications of L1, and thus of Alus and other sequences confined to simians, offer a plausible explanation for the lack of innovation in the trajectory of evolution in the prosimian lineages, compared with the innovation in the simian lineages. Since their divergence from the basal primates, the simians have experienced repeated periods of intense L1 activity that occurred from about 40 Mya to about 12 Mya [54]. The highly active simian L1s were responsible for the very large amplification of younger Alus and of many gene retrocopies [55]. Possibly, differential activity of the L1/Alu pair may have driven the trajectory and divergence of the simians, compared with the prosimians. The greater endogenization of some retroviruses in simians compared with prosimians [56] may also have played a part. These events may also explain the larger genome size of the simians compared with prosimians [57].

A significant feature of Alus is their dimeric structure, involving a fusion of two slightly dissimilar arms [58]. This added length and complexity seems to increase their effectiveness as a reservoir of evolutionarily useful DNA sequence or as an inducer of ectopic recombination. It may therefore be no coincidence that simian genomes are well endowed with dimeric Alus. Viable SINEs in the less fecund and less evolutionary innovative prosimians are heterogeneous, and include the conventional dimeric Alu, Alu-like monomers, Alu/tRNA dimers and tRNA SINEs [59]. This distinctly contrasts with simian SINEs; in simians, viable SINEs are almost entirely dimeric Alus. Thus, both qualitatively and quantitatively, the Alu dimer seems to represent a key example of the power of a SINE to strongly influence evolutionary trajectory.

Although these coincident events cannot, by themselves, be a clear indication of cause and effect, distinct Alu subfamilies (*AluJ, AluS, AluY*) correlate with the divergence of simian lineages [38,39]. Whereas the *AluJ *subfamily was active about 65 Mya when the separation and divergence between the simians and the prosimians occurred, the *AluS *subfamily was active beginning at about 45 Mya, when the Old World monkey proliferation occurred, followed by a surge in *AluY *activity and expansion beginning about 30 Mya, contemporaneous with the split between apes and Old World monkeys [38,39]. Thus, periodic expansions of Alu subfamilies in particular seem to correspond temporally with major divergence points in primate evolution. More recent Alu activity may be a factor in the divergence of the human and chimpanzee lineages, with Alus having been three times more active in humans than in chimpanzees [40,60]. Moreover, at least two new Alu subfamilies (*AluYa5 *and *AluYb8*) have amplified specifically within the human genome since the human-chimpanzee split [40,60,61].

Passive TE-Thrust mediated by the Alu/L1 pair has also been evident as a force contributing to lineage divergence in the primates. Ectopic recombinations between Alus, in particular, are a frequent cause of lineage-specific deletion, duplication or rearrangement. Comparisons between the human and chimpanzee genomes have revealed the extent to which they have passively exerted their effects in the relatively recent evolutionary history of primates. An examination of human-specific Alu recombination-mediated deletion (ARMD) identified 492 ARMD events responsible for the loss of about 400 kb of sequence in the human genome [62]. Likewise, Han *et al. *[63] reported 663 chimpanzee-specific ARMD events, deleting about 771 kb of genomic sequence, including exonic sequences in six genes. Both studies suggested that ARMD events may have contributed to the genomic and phenotypic diversity between chimpanzees and humans. L1-mediated recombination also seems to be a factor in primate evolution, with Han *et al. *[64] reporting 50 L1-mediated deletion events in the human and chimpanzee genomes. The observed high enrichment of TEs such as Alu at low-copy-repeat junctions indicates that TEs have been an important factor in the generation of segmental duplications that are uniquely abundant in primate genomes [39]. Such genomic duplications provide a major avenue for genetic innovation by allowing the functional specialization of coding or regulatory sequences. Karyotypic changes are thought to be an important factor in speciation [65]. Major differences between the human and chimpanzee genomes include nine pericentric inversions, and these have also been linked to TE-mediated recombination events [66]. It thus seems that both the active and passive effects of Alu and L1 have greatly facilitated and influenced the trajectory of simian evolution by TE-Thrust. Transfer RNA-type SINEs, with suitable partner LINEs, probably perform this role in other lineages.

## TE-Thrust affects evolutionary trajectory by engineering lineage-specific traits

TEs can act to generate genetic novelties and thus specific phenotypic traits in numerous ways. Besides passively promoting exon, gene or segmental duplications (or deletions) by unequal recombination, or by disruption of genes via insertion, TEs can actively contribute to gene structure or regulation via exaptation. On multiple occasions, TEs have been domesticated to provide the raw material for entire genes or novel gene fusions [11]. More frequently, TEs have contributed partially to individual genes through exonization after acquisition of splice sites [67,68]. Independent exons generated by TEs are often alternatively spliced, and thereby result in novel expressed isoforms that increase the size of the transcriptome [69]. The generation of novel gene sequences during evolution seems to be heavily outweighed by genetic or epigenetic changes in the transcriptional regulation of pre-existing genes [34,70]. Consistent with this, much evidence indicates that a major way in which TEs have acted to functionally modify primate genomes is by actively inserting novel regulatory elements adjacent to genes, thus silencing or enhancing expression levels or changing expression patterns, often in a tissue-specific manner [71-73]. Moreover, because they are highly repetitious and scattered, TEs have the capacity to affect gene expression on a genome-wide scale by acting as distributors of regulatory sequences or CpG islands in a modular form [74]. Many functional binding sites of developmentally important transcription factors have been found to reside on Alu repeats [75]. These include oestrogen receptor-dependent enhancer elements [76] and retinoic acid response elements, which seem to have been seeded next to retinoic acid target genes throughout the primate genome by the *AluS *subfamily [77]. As a consequence, TEs are able to contribute significantly to the species-specific rewiring of mammalian transcriptional regulatory networks during pre-implantation embryonic development [78]. Similarly, primate-specific ERVs have been implicated in shaping the human p53 transcriptional network [79] and rewiring the core regulatory network of human embryonic stem cells [80].

Certain classes of retro-TEs can actively generate genetic novelty using their retrotranspositional mechanism to partially or fully duplicate existing cellular genes. Duplication is a crucial aspect of evolution, which has been particularly important in vertebrates, and constitutes the primary means by which organisms evolve new genes [81]. LINEs and SVAs have a propensity to transduce host DNA due to their weak transcriptional termination sites, so that 3' flanking regions are often included in their transcripts. This can lead to gene duplication, exon shuffling or regulatory-element seeding, depending on the nature of the sequence involved [37,82,83]. Duplication of genes can also occur via the retrotransposition of mRNA transcripts by LINEs. Such genes are termed retrocopies, which, after subsequent useful mutation, can sometimes evolve into retrogenes, with a new, related function. There are reportedly over one thousand transcribed retrogenes in the human genome [84], with about one new retrogene per million years having emerged in the human lineage during the past 63 Myr [26]. Some primate retrogenes seem to have evolved highly beneficial functions, such as *GLUD2 *[37].

## Specific evidence for TE-Thrust: examples of traits engineered by TEs in the higher primates

**Figure 2:**
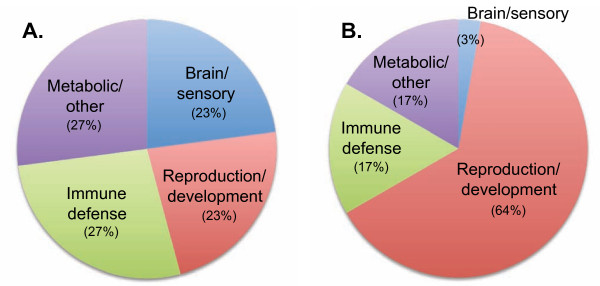
**Comparison of aspects of primate phenotype affected by **(A) **Alu elements and **(B) **LTR/ERVs. Based on the published data shown in Tables 3 to 6**.

TEs seem to have heavily influenced the trajectories of primate evolution and contributed to primate characteristics, as the simians in particular have undergone major evolutionary advancements in cognitive ability and physiology (especially reproductive physiology). The advancement and radiation of the simians seems to be due, in part and all else being equal, to exceptionally powerful TE-Thrust, owing to its especially effective Alu dimer, partnered by very active novel L1 families, supplemented by ERVs and LTRs. These have engineered major changes in the genomes of the lineage(s) leading to the simian radiations and major transitions. We identified more than 100 documented instances in which TEs affected individual genes and thus were apparently implicated at a molecular level in the origin of higher primate-specific traits (Table 3; Table 4; Table 5; Table 6). The Alu SINE dominated, being responsible for nearly half of these cases, with ERVs/sLTRs being responsible for a third, followed by L1-LINEs at 15% (Figure 1A). Just 2% were due to the young SVAs, and 1% each to ancient MIR SINEs and DNA-TEs. More than half the observed changes wrought by TEs were regulatory (Figure 1B). As discussed below, TEs seem to have influenced four main aspects of the primate phenotype: brain and sensory function, reproductive physiology, immune defence, and metabolic/other (Figure 1C and Table 3; Table 4; Table 5; Table 6). Notably, ERVs, which are often highly transcribed in the germline and placenta [85], were strongly associated with reproductive traits, whereas Alus influenced these four aspects almost equally (Figure 2).

### Brain and sensory function

The large brain, advanced cognition and enhanced colour vision of higher primates are distinct from those of other mammals. The molecular basis of these characteristics remains to be fully defined, but from evidence already available, TEs (particularly Alus) seem to have contributed substantially via the origination of novel genes and gene isoforms, or via altered gene transcription (Table 3). Most of the neuronal genes affected by TEs are restricted to the apes, and they seem to have roles in synaptic function and plasticity, and hence learning and memory. These genes include multiple neurotransmitter receptor genes and *glutamate dehydrogenase 2 *(*GLUD2)*, a retrocopy of *GLUD1 *that has acquired crucial point mutations. *GLUD2 *encodes glutamate dehydrogenase, an enzyme that seems to have increased the cognitive powers of the apes through the enhancement of neurotransmitter recycling [37]. The cell cycle-related kinase (*CCRK*) gene represents a good example of how the epigenetic modification of TEs can be mechanistically linked to the transcriptional regulation of nearby genes [86]. In simians, this gene possesses regulatory CpGs contained within a repressor Alu element, and these CpGs are more methylated in the cerebral cortex of human compared with chimpanzee. Concordantly, *CCRK *is expressed at higher levels in the human brain [86]. TEs may also affect the brain at a somatic level, because embryonic neural progenitor cells have been found to be permissive to L1 activity in humans [87]. This potentially provides a mechanism for increasing neural diversity and individuality. As our human lineage benefits from a diversity of additional individual talents, as well as shared talents, this phenomenon, if confirmed, could increase the 'fitness' of the human lineage, and is entirely consistent with the concept of differential survival of lineages, as stated in our TE-Thrust hypothesis.

The trichromatic vision of Old World monkeys and apes immensely enhanced their ability to find fruits and other foods, and probably aided them in group identity. This trait evidently had its origin in an Alu-mediated gene-duplication event that occurred about 40 Mya, and subsequently resulted in two separate cone photoreceptor (opsin) genes [36], the tandem *OPN1LW *and *OPN1MW*, which are sensitive to long- and medium-wave light respectively. Other mammals possess only dichromatic vision.

### Reproductive physiology

Compared with other mammals, simian reproduction is characterized by relatively long gestation periods and by the existence of a hemochorial-type placenta that has evolved additional refinements to ensure efficient fetal nourishment. Available data suggests that TE-Thrust has contributed much of the uniqueness of the higher primate placenta, which seems to be more invasive than that of other mammals, and releases a large number of factors that modify maternal metabolism during pregnancy. These characteristics appear to be due to the generation of novel placenta genes and to various TEs having been exapted as regulatory elements to expand or enhance the expression of pre-existing mammalian genes in the primate placenta (Table 4). The growth hormone (*GH*) gene locus is particularly notable for having undergone rapid evolution in the higher primates compared with most other mammals. A crucial aspect of this evolutionary advance was a burst of gene-duplication events in which Alu-mediated recombination is implicated as a driving force [88]. The simians thus possess between five and eight *GH *gene copies, and these show functional specialization, being expressed in the placenta, in which they are thought to influence fetal access to maternal resources during pregnancy [88,89]. Longer gestation periods in simians were accompanied by adaptations to ensure an adequate oxygen supply. One key event was an L1-mediated duplication of the *HBG *globin gene in the lineage leading to the higher primates, which generated *HBG1 *and *HBG2 *[90]. *HBG2 *subsequently acquired expression specifically in the simian fetus, in which it ensures the high oxygen affinity of fetal blood for more efficient oxygen transfer across the placenta. Old World primates additionally express *HBG1 *in the fetus, owing to an independent LINE insertion at the beta globin locus [91]. Thus, the important process of placental gas exchange has been extensively improved by TEs in simians, in contrast to that of many mammals, including prosimians, in which fetal and adult haemoglobins are the same.

Two prominent examples of functionally exapted genes whose sequences are entirely TE-derived are *syncytin-1 *(*ERVWE1*) and *syncytin-2 *(*ERVWE2*). Both of these primate-specific genes are derived from ERV envelope (*env*) genes [92,93]. The syncytins play a crucial role in simian placental morphogenesis by mediating the development of the fetomaternal interface, which has a fundamental role in allowing the adequate exchange of nutrients and other factors between the maternal bloodstream and the fetus. In a remarkable example of convergent evolution, which attests to the importance of this innovation, two ERV *env *genes, *syncytin*-*A *and *syncytin*-*B*, independently emerged in the rodent lineage about 20 Mya [94], as did *syncytin-Ory1 *within the lagomorphs 12-30 Mya, and these exhibit functional characteristics analogous to the primate syncytin genes [95]. This example, as well as many others (Table 3; Table 4; Table 5; Table 6) suggests the possibility that TE-Thrust may be an important factor in convergent evolution, a phenomenon that can be difficult to explain by traditional theories.

### Immune defence

Immune-related genes were probably crucial to the primate lineage by affording protection from potentially lethal infectious diseases. TEs have been reported to contribute to higher primate-restricted transcripts, or to the expression of a wide variety of immunologically relevant genes (Table 5). One example is the insertion of an AluY element into intron 1 of the fucosyltransferase (*FUT*)*1 *gene in an ancestor of humans and apes. This enabled erythrocytic expression of *FUT1*, and thus the ABO blood antigens [96], an adaptation linked to the selective pressure by malarial infection [97]. A particularly good example of a primate-specific adaptation that can be accounted for by a TE is the regulation of the cathelicidin antimicrobial peptide (*CAMP*) gene by the vitamin D pathway. Only simians possess a functional vitamin D response element in the promoter of this gene, which is derived from the insertion of an AluSx element. This genetic alteration enhances the innate immune response of simians to infection, and potentially counteracts the anti-inflammatory properties of vitamin D [98].

### Metabolic/other

TEs seem to underlie a variety of other primate adaptations, particularly those associated with metabolism (Table 6). A striking example, related to dietary change, was the switching of the expression of certain α-amylase genes (*AMY1A, AMY1B *and *AMY1C*) from the pancreas to the salivary glands of Old World primates. This event, which was caused by the genomic insertion of an ERV acting as a tissue-specific promoter [99], facilitated the utilization of a higher starch diet in some Old World primates. This included the human lineage, in which consumption of starch became increasingly important, as evidenced by the average human having about three times more *AMY1 *gene copies than chimpanzees [100]. Another example was the loss of a 100 kb genomic region in the gibbons, due to homologous recombination between AluSx sites [101], resulting in gibbons lacking the *ASIP *gene involved in the regulation of energy metabolism and pigmentation, which may help to account for their distinctive low body mass, so beneficial for these highly active arboreal primates.

## TE-Thrust and divergence of the human lineage

Human and chimpanzee genomes exhibit discernable differences in terms of TE repertoire, TE activity and TE-mediated recombination events [21,40,54,60-64]. Thus, although nucleotide substitutions to crucial genes are important [31], TE-Thrust is likely to have made a significant contribution to the relatively recent divergence of the human lineage [102,103]. In support of this, at least eight of the examples listed (Table 3; Table 4; Table 5; Table 6) are unique to humans. A notable example of a human-specific TE-mediated genomic mutation was the disruption of the *CMAH *gene, which is involved in the synthesis of a common sialic acid (Neu5Gc), by an AluY element over 2 Mya [104]. This may have conferred on human ancestors a survival advantage by decreasing infectious risk from microbial pathogens known to prefer Neu5Gc as a receptor.

## Conclusions

A role for TEs in evolution has long been recognized by many, yet its importance has probably been underestimated. Using primates as exemplar lineages, we have assessed specific evidence, and conclude that it points strongly to an instrumental role for TEs, via TE-Thrust, in engineering the divergence of the simian lineage from other mammalian lineages. TEs, particularly Alu SINEs, have essentially acted as a huge primate-restricted stockpile of potential exons and regulatory regions, and thereby have provided the raw material for these evolutionary transitions. TEs, including Alu SINEs, L1 LINEs, ERVs and LTRs have, through active TE-Thrust, contributed directly to the primate transcriptome, and even more significantly by providing regulatory elements to alter gene expression patterns. Via passive TE-Thrust, homologous Alu and L1 elements scattered throughout the simian genome have led to both genomic gain, in the form of segmental and gene duplications, and genomic loss, by promoting unequal recombination events. Collectively, these events seem to have heavily influenced the trajectories of primate evolution and contributed to characteristic primate traits, as the simian clades especially have undergone major evolutionary advancements in cognitive ability and physiology. Although as yet incompletely documented, the evidence presented here supports the hypothesis that TE-Thrust may be a pushing force for numerous advantageous features of higher primates. These very beneficial features apparently include enhanced brain function, superior fetal nourishment, valuable trichromatic colour vision, improved metabolism, and resistance to infectious-disease agents. Such large evolutionary benefits to various primate clades, brought about by various TE repertories, powerfully demonstrate that if TEs are 'junk' DNA then there is indeed much treasure in the junkyard, and that the TE-Thrust hypothesis could become an important part of some future paradigm shift in evolutionary theory.

## Abbreviations

ARMD: Alu recombination-mediated deletion; DNA-TE: DNA transposon; ERV: endogenous retrovirus; L1: LINE-1; LINE: long interspersed nuclear element; LTR: long terminal repeat; MIR: mammalian-wide interspersed repeat; Mya: million years ago; Myr: million years; retro-TE: retrotransposable element; RT: reverse transcriptase; SINE: short interspersed nuclear element; SVA: SINE-VNTR-Alu; TE: transposable element.

## Competing interests

The authors declare that they have no competing interests.

## Authors' contributions

KRO and WKG contributed equally to the writing and the research for this article. Both authors approved the final manuscript.
